# Improving Human Papillomavirus-Related Knowledge and Attitudes Among Ethnically Diverse Young Adults

**DOI:** 10.1089/heq.2018.0091

**Published:** 2019-05-28

**Authors:** Sharice M. Preston, William W. Darrow

**Affiliations:** ^1^ Department of Health Promotion and Behavioral Sciences, University of Texas Health Science Center at Houston School of Public Health, Houston, Texas.; ^2^ Department of Health Promotion and Disease Prevention, Robert Stempel College of Public Health and Social Work, Florida International University, Miami, Florida.

**Keywords:** HPV, HPV vaccination, knowledge, awareness, Hispanic, college, education, intervention

## Abstract

**Purpose:** To make baseline comparisons and evaluate the efficacy of an intervention designed to improve human papillomavirus (HPV) and HPV vaccine awareness, knowledge, and attitudes among ethnically diverse participants.

**Methods:** *Design:* Pre- and post-intervention surveys. *Setting:* An urban, Hispanic-Serving South Florida university. *Subjects:* Three hundred eighty-seven diverse college students attending a gender studies course. *Intervention:* Students received a brief educational message designed to improve HPV-related knowledge and attitudes. Baseline and follow-up survey data were collected. *Measures:* Outcome measures included baseline and follow-up awareness of HPV, perceived knowledge of HPV and HPV vaccination, measured knowledge score, vaccine attitudes, and doses of HPV vaccine received. *Analysis:* Chi-square, analysis of covariance (ANCOVA), and Wilcoxon rank-sum tests were used to compare baseline differences and evaluate the efficacy of the intervention.

**Results:** Hispanic participants had more positive attitudes toward HPV vaccination (62% vs. 44%, *p*=0.009) and were more willing to become vaccinated (66% vs. 46%, *p*=0.02) than non-Hispanic participants at baseline. Hispanic women (48%) were more likely to have initiated HPV vaccination than Hispanic men (27%, *p*=0.006). At baseline, only 30% of participants scored ≥4/7 points in knowledge. Participants' HPV knowledge improved by 41% after the intervention, with no difference by ethnicity in the post-intervention score.

**Conclusion:** The intervention was useful in improving HPV-related knowledge and attitudes among diverse college students. Future studies should examine barriers to vaccination among ethnic minorities.

## Purpose

Human papillomavirus (HPV) infection is the most common sexually transmitted infection globally and is responsible for multiple clinical outcomes, such as genital warts and cervical, anal, and oropharyngeal cancers.^1^ Safe and effective vaccines have been licensed to protect against HPV and have been proven to significantly decrease HPV-related morbidity in both men and women.^2^ The Advisory Committee on Immunization Practices recommends that boys and girls be vaccinated against HPV starting at 9 years old, with two doses over a 6-month period. Catch-up vaccination is recommended on a three-dose schedule for those older than 13 years who were not previously vaccinated at the recommended age of 9–26 years.^3^

Hispanics make up the largest ethnic minority group in the United States. Florida leads the country in number of residents of Cuban descent and is second behind New York in number of Puerto Ricans.^4,5^ Despite advancements in screening, the state of Florida has one of the highest cervical cancer death rates nationwide.^6^ Hispanics residing in the United States generally have lower rates of cancers compared to non-Hispanic whites, apart from cancers caused by an infectious agent, as is the case with cervical cancer and HPV.^7^ HPV-related knowledge is reportedly lower among Hispanic populations compared to non-Hispanic whites in the United States, as ethnic minorities are less likely to receive relevant vaccination recommendations from health care providers, in addition to perceiving a lower threat of HPV.^8,9^

Hispanic women overall have high rates of HPV infection and low rates of cervical cancer screening nationwide, leading to higher incidence of cervical cancer than non-Hispanic white and black populations.^10,11^ Hispanic men also have a higher incidence of HPV-associated penile cancer than non-Hispanic men.^10^

Puerto Ricans, who have high ethnic density in Florida, have consistently displayed high rates of cervical cancer among all Hispanic nationalities.^5^ Unlike other Hispanic subpopulations, Cubans living in the United States have lower cervical cancer rates that are similar to non-Hispanic whites. Significant variability in cancer rates exists between Hispanics of different countries of origins and contributes to the overall reported cervical cancer statistics of Hispanics in Florida. These distinct cancer profiles are reflective of cancer rates in the countries of origin.

Students attending college create a unique opportunity to access individuals with increased risk who may have missed opportunities to become vaccinated against HPV and are now of age to make decisions of their own regarding vaccination.^12^ Those aged 15–24 account for approximately half of all new infections every year, making this age bracket a priority population for catch-up vaccination.^13^ An early study found that of various sexually transmitted infections assessed, college students knew the least about HPV, with 37% of students never even hearing of the virus.^14^ Among female students who reported being diagnosed with HPV, 68% had not heard of the virus before being diagnosed. Men were less knowledgeable about HPV, despite their high prevalence of HPV infection.

HPV knowledge deficits among college students have not been resolved: a 2012 survey of college males aged 18–25 found that 74% of the participants had not received the HPV vaccination, and only 14.2% had completed the full series.^15^ Almost 50% of the respondents had never heard of HPV nor were they aware that there was a vaccine. Participants also had low awareness that men should be vaccinated against HPV and low awareness that they fell within the age bracket recommended for vaccination. These perceptions were common despite reporting low condom use and high numbers of lifetime sexual partners.

HPV educational interventions have been successfully used in various settings, primarily with women and parents.^16–19^ Interventions targeting college students have been successful in briefly, yet sustainably, delivering information designed to increase HPV knowledge and intentions to become vaccinated.^20–25^ Very few of these interventions study the potential impact of HPV education on knowledge and vaccine attitudes among Hispanic or ethnically diverse college students.

Recent studies have had mixed results in correlating Hispanic ethnicity with HPV vaccine acceptability, and many of these had limited participation of Hispanic men^26,27^ or targeted low-resource populations rather than college students.^8,9^ A 2016 study found that Hispanic college men in south Florida did not think that HPV was a risk to them or consider HPV vaccination a priority.^28^ None of these examined HPV vaccinations as a function of vaccine hesitancy, which the World Health Organization defines as a “delay in acceptance or refusal of vaccines despite availability of vaccination services.”^29^

Hispanic and other ethnic minority groups should be knowledgeable about the potential risks of HPV and the benefits that can accrue by becoming vaccinated against it. In support of the Healthy People 2020 goal to reach 80% HPV vaccine coverage,^30^ the aim of this study was to develop and then test the efficacy of an educational intervention for improving HPV-related knowledge and attitudes among ethnically diverse college students enrolled in a Hispanic-Serving Institution (HSI) in south Florida, a region known to have many Cuban and other Hispanic populations and a high incidence of cervical cancer.

## Methods

### Setting

The university that participants in this study attended is a large, urban, HSI with a largely Hispanic student population (currently 67% Hispanic of about 55,000) located in Miami, a large majority Cuban American city in Florida.^31^ HSIs have been defined as institutions in which at least “25% are full-time equivalent undergraduates who are citizens or resident aliens” of Hispanic origin.^32^ The university is known to have ethnic diversity that resembles Miami's, with over half of the city's residents being Hispanic and approximately a third of the residents being of Cuban descent.^33^

### Design

From April 2015 to February 2017, we conducted a brief in-class intervention in English with students enrolled at a public, urban HSI in south Florida. The university requires all students to speak, read, write, and understand English when enrolled. The intervention utilized a pre-test and post-test to evaluate the efficacy of a 30-min educational presentation and question and answer session designed to improve basic knowledge of HPV and HPV vaccination among college-aged young adults.

### Sample

The educational intervention was targeted to an ethnically diverse purposive sample of students enrolled at a large Hispanic-serving university situated in south Florida. We recruited from four undergraduate gender studies courses from the spring 2015 to spring 2017 semesters. In addition, we sampled from three dietetics and public health courses in January and February of 2017.

Every member of the seven classes who attended on the day of data collection was asked to participate (total enrollment of 409). Participants were read a brief statement regarding voluntary participation and consent, and students were permitted to leave the room at any point if they did not wish to participate. Students under the age of 18 were excluded from study participation. The university's institutional review board approved the study protocol before implementation.

### Measures

The self-administered written questionnaires elicited self-perceived and measured knowledge about HPV, as well as attitudes regarding severity, susceptibility, benefits, and barriers related to HPV and HPV vaccination. The matched pre- and post-intervention questionnaires consisted of a total of 62 items (38 in the pretest, 24 in the post-test). It was adapted, in part, from the 2009 University of North Carolina Men's Health Survey,^34,35^ with additional questions added after a review of the more recent literature. Surveys were pretested in a focus group and subsequently revised.

At baseline, demographic data (i.e., age, gender identity, ethnicity/race, health insurance status, sexual orientation, relationship status, sexual practices, and number of partners) were collected from participants. This was followed by a single question asking participants if they had ever heard of HPV before that day (yes, no, don't know). Participants then indicated how many doses of HPV vaccine they had received as of that day (0–3, don't know) and, subsequently, if they were not already fully vaccinated, if they were willing to take the HPV vaccine (yes, no, don't know). Participants were then asked to describe their self-perceived knowledge of HPV and HPV vaccines in two separate questions as “Nothing at all,” “a little,” “a moderate amount,” or “quite a lot.”

We measured actual HPV knowledge with seven true/false items; correct scores were totaled, and respondents were categorized as either high knowledge (four or more questions correct) or low knowledge (three or less questions correct). Eleven questions measured attitudes toward various HPV-related statements on a five-point Likert scale ranging from “strongly agree” to “strongly disagree” with an additional “don't know” option. All knowledge items were addressed in the educational portion of the intervention and further clarified if needed during the question and answer session. The post-test included follow-up knowledge and attitude questions identical to the pretest with additional questions regarding participants' experience with the intervention.

### Intervention

The intervention was designed to improve knowledge and attitudes regarding HPV and HPV vaccination. It was piloted in its entirety in April 2015. The intervention took from 40–50 min to complete and consisted of a pretest, moderated educational presentation, question and answer session, and an immediate post-test.

Pre- and post-questionnaires were distributed before delivery of the intervention, matched by identical numerical identifiers. Each survey took ∼5–10 min to complete and was collected immediately upon completion. The evidence-based presentation contained 26 PowerPoint slides and introduced general information about HPV, including virus subtypes, transmission, clinical outcomes, diagnoses, treatments, prognosis, and vaccination/prevention options.

Presentation messages were tailored for ethnically diverse young adults by incorporating physical Hispanic representation in intervention materials.^36^ This was intended to develop “felt targetedness” among participants, an important construct in health marketing that identifies the feeling of being the intentional recipient of the health information.^37,38^ The question and answer sessions were approximately 10 min long and allowed students to ask more specific, often situational questions, although many times the questions were meant to confirm information already presented. Students received tokens of appreciation at the end of data collection, such as pens, pencils, lip balm, and condoms sponsored by the university's student health center.

### Analysis

We report percentages for the complete sample and percentages (*n*/*N*) when analyses contain missing data. Univariate frequencies were calculated for demographic variables in the entire sample and then stratified by ethnicity (Hispanic vs. non-Hispanic). Survey questions were collapsed into dichotomous outcomes where appropriate. Attitude items were coded such that “strongly agree” or “agree” corresponded to a positive attitude toward that topic.

Pearson's Chi-square or Fisher's exact tests were performed to determine differences between and among Hispanic/non-Hispanic groups. Wilcoxon rank-sum tests characterized pre- versus post-intervention results. In addition, mean knowledge change from pretest to post-test was evaluated with paired sample *t* tests and one-way analysis of covariance (ANCOVA). Statistical significance was set at *p*<0.05. Reliability assessment of the knowledge score was conducted using Cronbach's alpha. All analyses were performed using Statistical Package for the Social Sciences (version 22.0).^39^

## Results

### Descriptive analyses

Of the 409 students consenting to participate, two were under the age of 18 and excluded from the study. Three hundred ninety-seven participants submitted post-intervention surveys (98%), seven of which did not include baseline data and were excluded. Of these, 382 reported an ethnic identity and were included in the current analysis. Participants were mostly female (71%), with an average age of 22 years (standard deviation [SD]=5.1). Over half were Hispanic (66%), white (61%), and single (66%).

 No statistical differences were evident between Hispanic and non-Hispanic participants regarding age, gender, sexual orientation, lifetime sexual experiences, relationship status, or insurance status (Table 1).

**Table 1.** Sociodemographic Characteristics of Participants by Ethnicity

	Total *N*=382	Hispanic *n*=252	Non-Hispanic *n*=129
Characteristics	Mean (SD)	Mean (SD)	Mean (SD)
Age, years	22.4 (5.0)	22.2 (4.5)	22.6 (5.4)

	***N***	***n* (%)**	***n* (%)**
Sexual orientation			
Straight	244	154 (61.4)	90 (70.3)
Gay/lesbian	66	48 (19.1)	18 (14.1)
Bisexual	53	36 (14.3)	17 (13.3)
Other	16	13 (5.2)	3 (2.3)
Race			
White	233	197 (77.9)	36 (27.9)
Black	82	15 (5.9)	67 (51.9)
Other	67	41 (16.2)	26 (20.2)
Lifetime sexual partners			
0	54	36 (14.3)	18 (14)
1–5	214	150 (59.8)	64 (49.6)
6–10	55	32 (12.7)	23 (17.8)
>10	57	33 (13.1)	24 (18.6)
Health insurance			
Insured	234	158 (79.4)	76 (74.5)
Uninsured	52	30 (15.1)	22 (21.6)
Don't know	15	11 (5.5)	4 (3.9)
Relationship status			
Single	254	162 (64.0)	90 (69.8)
Married	16	10 (4.0)	6 (4.7)
Divorced/widowed	4	2 (0.8)	2 (1.6)
In a relationship	110	79 (31.2)	31 (24.0)

SD, standard deviation.

### Baseline

#### Awareness and knowledge

Hispanic participants (86%) were more likely than non-Hispanic participants (78%) to have heard of HPV before the intervention (*p*=0.05). Non-Hispanic (63%) participants were more likely than Hispanic participants (54%) to report receiving at least some information about HPV before the intervention, although this difference was not significant at the 95% confidence level (*p*=0.06).

Twenty-one percent of Hispanic males had never heard of HPV before the intervention experience, and 58% reported receiving no information about HPV (similar to non-Hispanic men) compared with 41% of Hispanic women (*p*=0.02). When asked “How much would you say you know about HPV?” about a third of Hispanic and non-Hispanic participants described their knowledge as “moderate” or “a lot.” Regarding HPV vaccination, 32% of participants reported moderate-to-high self-perceived knowledge. At baseline, 70% of all participants received low scores (<4/7) on HPV knowledge, with no difference by ethnicity.

#### Vaccine uptake

**FIG. 1.**
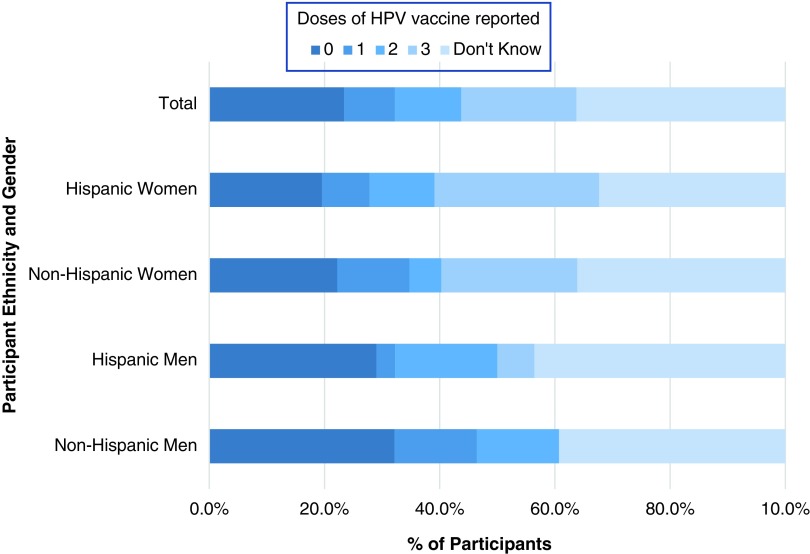
Human papillomavirus vaccine initiation and completion rates by gender and ethnicity.

While 41% of respondents reported receiving at least one dose of HPV vaccine, only 20% of respondents had received three doses (Fig. 1). Forty-one percent of Hispanic participants reported having one or more doses of HPV vaccine, a similar percentage as non-Hispanic participants (39%, *p*>0.05). Hispanic women (48%) were more likely than Hispanic men (27%) to have received at least one dose of HPV vaccine (*p*=0.006), but non-Hispanic men and women were equally likely to report receiving at least one dose (*p*>0.05).

#### Vaccine attitudes

Regardless of ethnicity, 69% of participants reported that HPV was a serious concern for women, while only 47% reported that HPV was a serious concern for men. Hispanic men were significantly more likely than Hispanic women to be unsure regarding many of the attitude questions. Fifty-seven percent of Hispanic men reported being unsure if HPV was a rare infection. Despite this, 39% of Hispanic men were unsure if HPV was a serious concern for women, while 49% were unsure about the threat to men.

The majority of participants (72%) reported positive attitudes toward general vaccine safety and effectiveness. Hispanic students (62%) were more likely to have a positive opinion at baseline of the HPV vaccine (vs. 44%, *p*=0.009), and more likely (66%) than non-Hispanic students (46%) to be willing to take the HPV vaccine if not already vaccinated (*p*=0.02). Hispanic students were more likely than non-Hispanic students to report that women (74% vs. 62%, *p*=0.04) and, to a lesser extent, men (68% vs. 58%, *p*=0.04) should be vaccinated against HPV.

Twelve percent of participants who agreed that women should be vaccinated against HPV disagreed or were unsure if men should be vaccinated. Two-thirds of Hispanic participants (67%) agreed with the statement that “everyone should be vaccinated against HPV” compared with 55% of non-Hispanic participants (*p*=0.04). Non-Hispanic men (37%) were less likely to believe that everyone should be vaccinated against HPV than non-Hispanic women (61%, *p*=0.03).

### Post-intervention

#### Knowledge

**FIG. 2.**
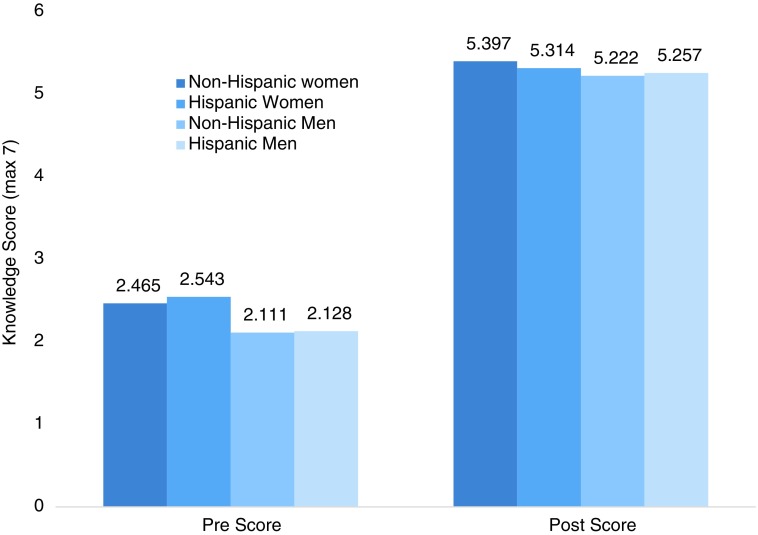
Change in mean knowledge score from baseline to post-intervention by ethnicity and gender.

Immediately after the intervention, participants showed a statistically significant improvement in knowledge and attitudes toward HPV and vaccination. When asked again about their self-perceived knowledge of HPV, 94% of those who felt limited knowledge at baseline felt that they knew more following the intervention. Similarly, 91% felt more knowledgeable about the HPV vaccines following the intervention. Participants improved their average knowledge score from 2.4 points (SD=2.0) to 5.3 (SD=1.5) out of seven (*p*<0.001; Fig. 2). There was no difference in post-intervention knowledge score between ethnicities after means were adjusted for preintervention score F(1, 349)=0.276, *p*=0.60.

Further stratification by gender showed that men, regardless of ethnicity, performed just as well as women on the post-test despite earning slightly lower baseline knowledge scores (Fig. 2). Individual item analysis showed that participants significantly improved in each knowledge measure except one. At post-test 46% of participants still incorrectly answered that herpes was caused by HPV, despite this being an improvement from 83% incorrect at baseline.

#### Vaccine attitudes

Consideration of HPV as a serious concern for women increased from 69% at baseline to 92% (*z*=−10.2, *p*<0.001) and from 47% to 86% for men (*z*=−8.2, *p*<0.001). Despite this, only 63% of women and 73.6% of men felt that HPV was a threat to their own health. Hispanic participants were still more likely than non-Hispanic participants to agree that women (93% vs. 86%, respectively, *p*=0.05) and men (92% vs. 86%, respectively, *p*=0.05) should be vaccinated against HPV.

Attitudes toward general vaccine safety and effectiveness moderately increased following the intervention (from 72% in pretest to 81%). Opinions of the HPV vaccine improved overall at follow-up, with non-Hispanic attitudes improving to 73%, yet not enough to close the significant attitude gaps seen at baseline compared to Hispanic participants (81%, *p*=0.04).

#### Intervention evaluation

No differences were found in evaluating components of the intervention. Both Hispanic and non-Hispanic participants found the intervention experience to be highly acceptable; 93% rated the overall intervention experience to be “positive” or “very positive.” When assessing each component of the intervention, 96% of participants found the educational presentation to be an appropriate length. Ninety-eight percent felt the question and answer session that followed the educational portion to be useful, and 97% felt that they learned a great deal from their participation.

## Discussion

This study evaluated the differences in intervention effects between Hispanic and non-Hispanic students regarding HPV and vaccination, with special attention paid to attitudes toward the safety and effectiveness of vaccines in general as a proxy measure of vaccine hesitancy. College students aged 18–26 are in a unique position to participate in catch-up HPV vaccination and should be educated on HPV and vaccination options. A brief HPV educational intervention administered to ethnically diverse, undervaccinated young adults can potentially improve vaccination rates. HPV knowledge and attitudes can significantly be altered; gender and ethnic disparities can be dramatically reduced.

Baseline knowledge of HPV and HPV vaccination was equally low in our sample of undergraduate students. Women had similar baseline knowledge of HPV and HPV vaccination as their male counterparts, which was a surprising finding, given that the vaccine was initially intended and marketed for women and girls.^40^ Several studies find women surpassing men in HPV-related knowledge.^41,42^ Vaccine initiation (≥1 dose) in Hispanic students was 41%, similar to non-Hispanics in this sample, despite reporting receiving less HPV-related information before involvement in the intervention. Hispanic adolescents surveyed in a 2015 Florida study reported 61.7% of women and 50.1% of men having received at least one dose of HPV vaccine,^43^ a higher coverage than observed in our study.

Results at our university fall well below national HPV vaccine coverage of 63% for adolescent women and far below the 80% coverage rate needed to see real reductions in the medical burden of HPV. This is most likely because students attending college from 2015 to 2017, when this study was conducted, were most likely not vaccinated against HPV as a child due to completing routine vaccinations before the release of the updated recommendations. In addition, boys and men were not added to the HPV vaccination recommendations until 2011.

Despite finding similar rates of vaccine initiation in Hispanic and non-Hispanic participants in our study, there were significant differences in the way Hispanic participants viewed HPV vaccination compared to non-Hispanic participants, which extended into differences in willingness to become vaccinated in the future. Several studies describe Hispanic vaccine consumers as being wary of side effects,^44,45^ although we found Hispanic students to have more positive views of HPV vaccination. This may be explained by a burgeoning hesitancy to vaccines among non-Hispanic whites, who rely less on the medical opinions of doctors than Hispanics to help them make vaccination decisions.^46^

Hispanic and non-Hispanic participants evaluated the components of the intervention as very positive. Nevertheless, in 10% of non-Hispanic cases (vs. 5% in Hispanic cases), participant attitudes were even more negative post-intervention than at baseline, indicating that the intervention may have reinforced existing vaccine-hesitant attitudes among some students instead of counteracting them.

Hispanic participants were more likely to believe that HPV is a serious concern and expressed more willingness to become vaccinated if not already. Nevertheless, Hispanic positive attitudes toward vaccination in general, as well as toward HPV vaccine, may not be enough to influence vaccine coverage rates among Hispanics in Florida.

Hispanic participants also reported receiving less information on the topic of HPV and HPV vaccination, even with similar insurance coverage as non-Hispanic participants. In addition, Hispanic participants had more positive views of the safety and effectiveness of vaccination in general. These observations suggest that Hispanic students may have higher vaccine coverage than non-Hispanic students if they were provided with the amount of information that non-Hispanic students reported receiving before the intervention.

Many studies cite provider recommendation barriers such as time constraints, low perceived threat to the patient, and even preferential vaccination recommendations.^47^ With the established importance of receiving a provider recommendation for HPV vaccination, health care providers may benefit from increased training on inclusive education of all vaccine-eligible patients regardless of ethnicity.

There were several limitations in this study. It was conducted at a single HSI with students enrolled in a limited number of classes. Enrolled students may not be representative of the community or the population of college students in south Florida. Results of this study should not be generalized to other universities or the south Florida community without due caution. We recruited from a region known to have a large Cuban population. While we solicited optional qualitative information regarding specific nationality, few students offered this information, not allowing meaningful ethnic subgroup analyses. The university does not currently measure country of origin among noninternational students, so although participants identified themselves primarily as Hispanic, we cannot specify our findings to Cuban or other Hispanic populations due to sociodemographic and other differences between Cubans and other Hispanic nationalities.

Our assessments were conducted at baseline and immediately post-intervention, so sustainable changes in knowledge, favorable attitudes, and vaccine acceptance were not determined. Vaccination status was not assessed again in the weeks after the intervention, so we were unable to make any claims that the intervention prompted undervaccinated participants to become vaccinated. However, due to the brevity of the intervention, post-intervention assessments were completed within an hour of the baseline assessments, creating high certainty that factors other than the intervention did not influence or enhance the estimated effect of the intervention.

Despite these limitations, the intervention proved to be an excellent method for quickly improving knowledge and changing attitudes about HPV and HPV vaccination among diverse college students. The measures used to evaluate knowledge and attitudes demonstrated strong internal consistency. The intervention itself was inexpensive and easy to implement, making potential widespread use and adaptation feasible.

Our findings in this study suggest that consistent information provision may be a singular solution for a widespread problem. Intervention in the college setting has the potential to close disparities in HPV knowledge between ethnicities, as well as bolster vaccine attitudes and vaccination rates. Addressing HPV knowledge outside of a traditional clinical setting takes some of the burden off health care providers, who are restricted in time and sometimes resources to thoroughly educate patients about HPV. Comparison groups could be utilized in future studies to determine the comparative efficacy of a brief educational intervention to other prevention strategies.

Future studies assessing the role of education on HPV knowledge and attitudes may consider including increased measures on ethnicity and preexisting vaccination stances so that factors related to vaccine intentions can be thoroughly assessed. Particular care can be taken in future intervention studies to tailor HPV educational messages for Hispanic young men and women in both clinical and nonclinical settings, although our study found that simply providing the information may be sufficient among Hispanic college students.

Evidence points to provider recommendation as the single strongest event in the behavioral path to vaccination, with adolescents who received a recommendation five times more likely to receive a vaccination.^48^ Providers, however, are less likely to provide recommendations to racial/ethnic minorities. Future studies which assess attitude and prejudicial predictors of HPV vaccination recommendations among health care providers would contribute to this line of inquiry. Medical education programs should also develop HPV and HPV vaccination educational modules to increase provider confidence in giving strong vaccination recommendations, especially in response to hesitant attitudes. Providers should prioritize young adults during routine health screenings and work to minimize missed opportunities for vaccination in a culturally competent and inclusive manner.

Further intervention is necessary for certain populations and at all ecological levels—individual, community, and policy—to alleviate the burden of HPV infection and reduce HPV-related cancers. Public health professionals in education, academic, governmental, and nongovernmental sectors may increase multidisciplinary collaborative efforts to achieve such impact As financial barriers to vaccination decrease due to policy and nongovernmental efforts,^47^ attention should be turned to address nonfinancial barriers, such as lack of HPV knowledge, hesitant vaccine attitudes, and weak or optional provider recommendations. As the topic of HPV vaccination advances in public scrutiny, HPV interventions should now turn their focus to populations disproportionately burdened by HPV and HPV-related outcomes and populations at risk for underutilization of the vaccine.

## Conclusions

HPV-related knowledge and attitudes can be improved through a brief, tailored, and visually inclusive educational intervention experience in a college setting. Differences between Hispanic and non-Hispanic students at baseline disappeared at post-test, suggesting that educational intervention is an impactful method for addressing knowledge disparities in ethnic minority communities. Our findings also highlight the ongoing implications of vaccine-hesitant attitudes, despite the proven safety and effectiveness of HPV vaccines. While our approach was successful in bridging a gap for participants with vaccine-hesitant attitudes, more comprehensive strategies might be needed in the future to reach 80% HPV vaccination rates nationwide.

## Abbreviations Used

ANCOVA: analysis of covariance
HPV: human papillomavirus
HSI: Hispanic-Serving Institution
SD: standard deviation
